# Association between workload, compassion fatigue and presenteeism among maternal and newborn health professionals: the moderated role of self-compassion

**DOI:** 10.3389/fpsyg.2026.1793586

**Published:** 2026-06-15

**Authors:** Li Fu, Mingna Zhang, Na Wang, Lili Song, Jie Lu, Qi Fan, Yifei Yang, Luyi Liu, Sen Li, Dan E, Jenny Gamble, Debra Creedy

**Affiliations:** 1 School of Nursing, Liaoning University of Traditional Chinese Medicine, Shenyang, China; 2 School of Nursing, Capital Medical University, Beijing, China; 3 Beijing Obstetrics and Gynecology Hospital, Beijing Maternal and Child Health Care Hospital, Capital Medical University, Beijing, China; 4 Peking University People’s Hospital, Beijing, China; 5 Beijing Friendship Hospital, Capital Medical University, Beijing, China; 6 Monash University School of Nursing and Midwifery, Frankston, VIC, Australia; 7 School of Nursing and Midwifery, Griffith University, Logan, QLD, Australia

**Keywords:** compassion fatigue, cross-sectional survey, maternal and newborn professionals, presenteeism, self-compassion, workload

## Abstract

**Background:**

Presenteeism is prevalent in healthcare settings and is significantly associated with impaired physical and psychological wellbeing among healthcare professionals, as well as reduced quality of care. While workload is a known trigger, the psychological mechanisms linking it to presenteeism remain underexplored. Compassion fatigue may serve as a mediator, and self-compassion as a potential moderator, yet empirical evidence in maternal and newborn health settings is limited. Therefore, we aimed to investigate the roles of compassion fatigue and self-compassion on workload and presenteeism among maternal and newborn health professionals.

**Methods:**

A cross-sectional study was employed between October 2024 and June 2025. A total of 737 maternal and newborn health professionals from 13 hospitals in Beijing, China, completed measures of workload (NASA Task Load Index, NASA-TLX), presenteeism (Stanford Presenteeism Scale, SPS-6), compassion fatigue (Compassion Fatigue Short Scale, CF-Short Scale), and self-compassion (Self-Compassion Scale Short Form, SCS-SF). Data were analyzed using correlation, mediation, and moderated mediation analyses (PROCESS macro).

**Results:**

The mean presenteeism score among MNH professionals was 15.13 ± 4.21. Presenteeism was positively associated with workload and compassion fatigue, and negatively associated with self-compassion (all *p* < 0.01). Compassion fatigue mediated the relationship between workload and presenteeism (indirect effect B = 0.04, 95% CI [0.03, 0.06]). Self-compassion moderated both the direct effect of workload on compassion fatigue (B = −0.02, *p* < 0.01) and the indirect effect of workload on presenteeism via compassion fatigue (moderated mediation index = −0.002, 95% CI [−0.003, −0.001]), with higher self-compassion attenuating these relationships.

**Conclusion:**

Elevated workload increases presenteeism primarily through compassion fatigue. Importantly, self-compassion substantially mitigated this pathway, reducing associated fatigue risk and weakening its impact on work functioning. Interventions targeting workload management and the cultivation of self-compassion may help reduce presenteeism and support workforce wellbeing in MNH settings. However, the cross-sectional design of this study limits causal interpretation, and future longitudinal research is needed to confirm these pathways.

## 1 Introduction

Maternal and newborn health (MNH) professionals, including obstetric-gynecologic physicians and nurses, midwives, and maternity care assistants, play an indispensable role in facilitating respectful maternal care and safeguarding the health and wellbeing of women and newborns (Qiao et al., 2021; World Health Organization [WHO], 2018). However, MNH professionals routinely operate under substantial psychological demands, complex clinical responsibilities, and intense family interactions while caring for highly vulnerable populations (Cull et al., 2020). Such working conditions place them at heightened risk of presenteeism, defined as attending work despite health problems that impair performance, a behavior linked to deteriorated psychological wellbeing, psychological exhaustion, and reduced work effectiveness (Pereira et al., 2022). A growing body of international literature confirms that health professionals are highly susceptible to presenteeism (Madrazo et al., 2025) with a recent meta-analysis reporting a global prevalence of 49% among nurses (Min et al., 2022). In China, similar patterns are evident: 30.7% of physicians reported working while ill, often in addition to substantial symptoms of burnout (Pei et al., 2020). Notably, healthcare professionals working in maternal and newborn health settings demonstrated even higher vulnerability, with a reported prevalence of 55.3%, potentially due to the sustained psychological and emotional demands inherent in their professional roles (Taylor et al., 2022). Especially in China, cultural values emphasizing self-sacrifice, and loyalty to colleagues (Andrews et al., 2020) may reinforce a “push-through” mentality that normalizes working while unwell, thereby introducing significant risks. Similarly, a national survey in the United States found that, among obstetrics and gynecology residents and program directors, concerns about burdening colleagues and a sense of responsibility towards patients were the most common reasons for presenteeism (Sun et al., 2025). Despite the high prevalence, research exploring the psychological mechanisms underlying presenteeism in maternal and newborn healthcare professionals remains limited.

Presenteeism not only endangers personal psychological and physical health but also compromises care quality, increases clinical errors, reduces productivity and incurs substantial economic costs (Pereira et al., 2022; Rainbow, 2019). These consequences are especially concerning for MNH professionals, whose roles require continuous clinical judgment, close contact with vulnerable populations, and a commitment to woman-centered, family-focused care (Cull et al., 2020). In this context, working while unwell may result in delays in fetal monitoring, missed steps in labor support, or slower responses during obstetric emergencies, potentially jeopardizing maternal and neonatal safety (Jiang et al., 2023). These adverse effects may translate into direct productivity losses and additional hidden costs at the organizational level. For example, the annual economic loss attributable to presenteeism is estimated to be approximately 4.38 billion yuan in China, highlighting its substantial financial burden (Zhang et al., 2025). Given their pivotal role and unique vulnerabilities, understanding the mechanisms driving presenteeism in this workforce is essential to developing effective prevention and intervention strategies.

Among various occupational stressors, workload is recognized as a primary predictor of presenteeism (Gillet et al., 2020). In China, inefficient and uneven human resource allocation in maternal and child health services exacerbate this issue (Zhu et al., 2025). For instance, the ratio of midwives to pregnant women in China is approximately 1:4,000, far below the WHO standard of six midwives per 1,000 births, highlighting a critical personnel shortage (Hu, 2020). This shortage intensifies workloads, extends working hours, and limits leave opportunities, thereby elevating presenteeism (Pereira et al., 2022). Another study from Brazil also pointed out that here is a relationship between workloads and nursing presenteeism, verified by limitations and loss of productivity (Carvalho et al., 2021). Compared with general healthcare staff, MNH professionals often face more acute and time-sensitive clinical demands, particularly in obstetric emergencies such as postpartum hemorrhage or fetal distress, where rapid decision-making and continuous monitoring are essential. These high-acuity conditions introduce distinct stressors, including urgency, unpredictability, and the need to manage simultaneous risks to both mother and infant, which may further amplify the impact of workload on presenteeism (Jiang et al., 2023). Furthermore, the majority of MNH professionals are female, who often carry additional family responsibilities such as child-rearing and household management in the context of China (Zhang et al., 2025). Excessive workloads heighten the risk of work–family imbalance, physical and mental health problems, and diminished capacity to engage fully in professional tasks (Broetje et al., 2020). Given that presenteeism can pose many serious consequences, elucidating the mechanism linking workload to presenteeism is crucial.

Based on Conservation of Resources (COR) theory, individuals in high-demand environments expend emotional, attentional, and psychological resources to meet work demands and protect existing resources (Hobfoll, 1989). Continuous depletion of these resources can trigger a loss spiral, culminating in resource exhaustion (Bakker and Demerouti, 2024). In MNH settings, this process may be further intensified by occupation-specific physiological and clinical demands. Prolonged standing during labor, high-intensity care during emergency deliveries, and frequent night shifts can lead to cumulative physical fatigue, musculoskeletal strain, and sleep disruption (Matthews et al., 2024). Prolonged high workload and chronic physical strain may manifest as declining health, psychological exhaustion, and secondary traumatic stress — a constellation of symptoms known as compassion fatigue, which in turn increases the likelihood of presenteeism (Zhong et al., 2024). The prevalence of this condition is alarming. A systematic review reported that 77.6% obstetric and gynecological nurses experienced compassion fatigue (Lv et al., 2025). This vulnerability is compounded by the high-intensity clinical tasks they may be required to perform, as well as the provision of compassionate care. A substantial majority (71%–96.9%) of maternity health professionals are exposed to traumatic birth events (e.g., postpartum hemorrhage, neonatal asphyxia) (Uddin et al., 2022). Such exposure can exacerbate compassion fatigue, negatively impact professionals’ psychological wellbeing and emotional functioning, while also diminish caregiving capacity, concentration, and the overall quality of care (Zhang and Dator, 2025).

In this context, self-compassion — defined as treating oneself with kindness and support during suffering or stress — emerges as a critical personal resource that promotes psychological wellbeing (Neff, 2023). Aligned with the Job Demands-Resources (JD-R) model, which posits that job resources fuel motivation and buffer against strain (Demerouti et al., 2001), self-compassion can function as a positive psychological resource. Empirical evidence supports this perspective. A study among community nurses in the United Kingdom found that higher levels of self-compassion are associated with lower burnout, while greater compassion satisfaction is linked to higher empathy and lower occupational burnout (Durkin et al., 2016). A mixed study in Australia also showed that self-compassion increases the wellbeing of maternal and child healthcare workers and suggested that caregivers should care for themselves before caring for others (Steen et al., 2025). Evidence also suggested that self-compassion was significantly associated with psychological health among midwives following exposure to traumatic childbirth (Musseri Navon et al., 2025). Thus, the ability for self-compassion will help individuals manage occupational stress, reduce burnout, and sustain work engagement. For example, after traumatic events, MNH professionals often struggle with guilt and self-blame, which can erode empathy toward patients and themselves (Xu et al., 2025). Higher levels of self-compassion enable better emotional regulation, self-comfort, and maintained wellbeing, thereby supporting sustained work engagement (Kotera et al., 2024). Consequently, when faced with excessive workload, MNH professionals with greater self-compassion may be better protected from resource depletion that impacts compassion fatigue and subsequently presenteeism. Therefore, fostering self-compassion represents a promising strategy to mitigate adverse outcomes (Neff, 2023).

Integrating these perspectives, this study employed a moderated mediation analysis, to examine the relationship between workload and presenteeism among MNH professionals, with compassion fatigue as the mediator and self-compassion as the moderator. This approach aims to elucidate how psychological resources can be leveraged to interrupt the detrimental pathway to presenteeism, ultimately supporting the health of MNH professionals and the quality of care they provide.

**FIGURE 1:**
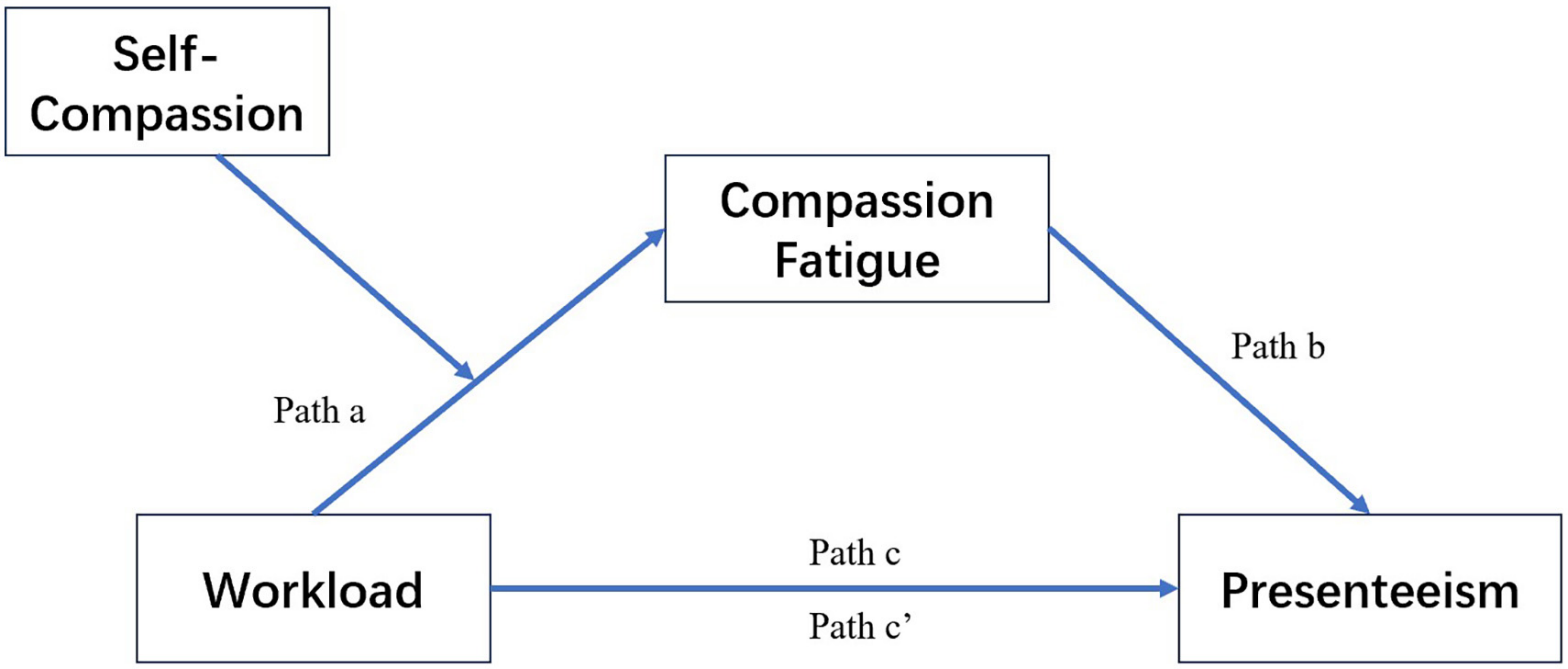
The hypothetical model.

As illustrated in Figure 1, we propose the following hypotheses based on the COR theory and JD-R model:

H1: Workload is positively correlated with presenteeism.

H2: Compassion fatigue mediates the relationship between workload and presenteeism.

H3: Self-compassion moderates the relationship between workload and compassion fatigue.

H4: Self-compassion moderates the indirect effect of workload on presenteeism through compassion fatigue (i.e., moderated mediation).

## 2 Materials and methods

### 2.1 Study design

A multicenter, cross-sectional online survey was conducted between October 2024 and June 2025 using convenience sampling. The reporting of this study was guided by STrengthening the Reporting of OBservational studies in Epidemiology guidelines (von Elm et al., 2014).

### 2.2 Participants and setting

Participants were licensed frontline MNH professionals —including midwives, obstetric-gynecologic clinicians, and yuesao (a culturally specific maternal-newborn health support worker in China) between October 2024 and June 2025. Thirteen participating sites comprised general tertiary hospitals as well as specialized maternal and child health hospitals, located across both urban and suburban districts in Beijing. Participants were included if they: (1) were aged over 18 years; (2) actively engaged in maternal and newborn health services; (3) had at least 1 year of work experience; and (4) had access to an electronic device and the internet. Participants were excluded if they were: (1) currently in training or pursuing advanced study;(2) on leave or vacation during the survey period, and/or (3) experiencing severe physical or mental illness.

### 2.3 Measures

The survey consisted of a demographic form and four standardized measures previously tested for use in the Chinese language. All questionnaires were pilot-tested among six MNH professionals who were not included in the final sample prior to the formal survey.

#### 2.3.1 Demographic characteristics

Demographic information included participants’ age, gender, ethnicity, education, marital status, number of children, employment type, monthly income, institution type, weekly night shifts, position role, work model, weekly work hours, and years of experience in the current position.

#### 2.3.2 NASA Task Load Index (TLX)

Workload was assessed using the NASA Task Load Index (NASA-TLX) (Hart and Staveland, 1988). The NASA-TLX contains six items: mental demand, physical demand, temporal demand, performance, effort, and frustration. Each item is rated on a 10-cm visual analog scale and converted to a 0–20 scale, with higher scores indicating greater workload. The Chinese version of the NASA-TLX scale was validated by Liang et al. (2019), with a reported Cronbach’s alpha coefficient of 0.71, a test-retest reliability of 0.806, indicating acceptable reliability. Exploratory factor analysis (EFA) showed a cumulative variance contribution rate of 62.615%, the scale-level content validity index (S-CVI/Ave) was 1.00, supporting good structural and content validity. Overall, the scale has demonstrated good reliability and validity in Chinese health workers.

#### 2.3.3 Chinese version of the Stanford Presenteeism Scale (SPS-6)

Presenteeism was assessed using the Chinese version of the Stanford Presenteeism Scale (SPS-6). The original scale was developed by Koopman et al. (2002) to assess presenteeism and productivity loss and was culturally adapted for Chinese populations by Zhao et al. (2010) who reported a high reliability and validity. The scale consists of six items rated on a five-point Likert scale from 1 (strongly disagree) to 5 (strongly agree). Total scores range from 6 to 30, with higher scores indicating a greater level of presenteeism. The Chinese version of SPS-6 demonstrated Cronbach’s α coefficients ranging from 0.76 to 0.90, indicating good reliability. Exploratory factor analysis showed a Kaiser-Meyer-Olkin (KMO) measure of 0.745, a significant Bartlett’s test of sphericity (*p* < 0.01), and a cumulative variance contribution of 81.3%, supporting the scale’s structural validity in Chinese populations.

#### 2.3.4 Chinese version of the Compassion Fatigue Short Scale (C- CF- Short Scale)

The Chinese version of the Compassion Fatigue Short Scale (C- CF- Short Scale), was originally developed by Adams et al. (2006) and later adapted for Chinese medical workers and firefighters by Sun et al. (2016). The scale consists of 13 items rated on a 10-point Likert scale (1 = rarely/never, 10 = very often), and comprises two dimensions: Secondary Stress (five items) and Job Burnout (eight items). Total scores range from 13 to 130, with higher scores indicating higher levels of compassion fatigue. The scale demonstrated good internal consistency among Chinese medical workers, with Cronbach’s alpha coefficients ranging from 0.87 to 0.90. Exploratory factor analysis showed that all factor loadings exceeded 0.50, with Bartlett’s test of sphericity significant (*p* < 0.001) and a KMO value of 0.849. Confirmatory factor analysis indicated a satisfactory fit for the two-factor model (CFI = 0.98, SRMR = 0.06, RMSEA = 0.07), supporting good structural validity. Overall, the Chinese version of the C-CF-Short Scale demonstrated good psychometric properties and is suitable for assessing compassion fatigue among Chinese medical workers.

#### 2.3.5 Self-Compassion Scale Short Form (SCS-SF)

Self-compassion was measured using the Self-Compassion Scale Short Form (SCS-SF) (Raes et al., 2011). The 12-item scale covers six two-item dimensions: self-kindness, self-judgment, common humanity, mindfulness, isolation, and over-identification. Each item is rated on a five-point Likert scale (1 = never always, 5 = almost always). After reverse-scoring relevant items, total scores range from 12 to 60, with higher scores indicating greater self-compassion. The scale demonstrated satisfactory internal consistency, with Cronbach’s alpha ≥ 0.86 in all samples. Confirmatory factor analysis indicated good model fit (CFI = 0.97, SRMR = 0.077, RMSEA = 0.080), and SCS-SF total scores were highly correlated with the full SCS (*r* ≥ 0.97), supporting its construct validity.

### 2.4 Sample size calculation

The rule of thumb for sample size calculation in structural equation modeling was applied in this study, with each estimated parameter requiring at least 10–20 observations (Rex, 2011). The proposed model was a first-stage moderated mediation model including four main variables, resulting in 12 estimated parameters. Based on a criterion of 20 participants per parameter, the minimum required sample size was 240.

### 2.5 Data collection

This survey was administered using the online platform “wen-juan-xing”.^1^ A project liaison officer was designated at each participating hospital to disseminate study information, recruit participants, and oversee data collection. Potential participants received the survey link or QR code from the liaison officer and accessed the questionnaire by scanning or clicking the link. The first page provided an overview of the study’s aims and procedures and provided ethical information (such as anonymity, confidentiality, and right to withdraw at any time) before requesting consent. The system restricted each respondent to a single submission to prevent duplicate entries. After downloading data from the platform, responses were screened for quality; and excluded if the completion time was under three minutes or if they contained clear inconsistencies.

### 2.6 Statistical analysis

Prior to analysis, the dataset underwent thorough cleaning. Responses that did not meet the inclusion and exclusion criteria, duplicate or invalid responses, and records with incomplete data were removed. The remaining data were checked for logical consistency to ensure validity, resulting in 737 valid samples.

Statistical analyses were performed using SPSS PEOCESS version 5.0 and R version 4.5.0. Descriptive statistics summarized participants’ demographic characteristics, with categorical variables presented as frequencies and percentages, and continuous variables reported as means ± standard deviations (SD). Missing values were handled before analysis. Missing values for continuous variables were filled with the mean (or median) of the variable, while missing values for categorical variables were filled with the mode because of the low missing value rate (<5%). We conducted a normality test before data analysis and selected appropriate statistical analysis methods based on the test results.

Univariate analysis was conducted to identify any significant differences in total Presenteeism scores across demographic variables. Pearson correlation analysis was used to examine relationships among key variables. Mediation (Model 4) and moderated mediation analyses (Model 7) were performed using the SPSS PROCESS macro, with marital status, children, employment status, institution type treated as confounders. Multicollinearity was assessed prior to the analysis. Tolerance values ranged from 0.4 to 0.8 and VIF values were all below 3, indicating no evidence of multicollinearity. A simple slope test was conducted to investigate the association between workload and compassion fatigue at different levels of self-compassion (low: M−1SD; high: M+1SD). Continuous variables were mean-centered prior to analysis to reduce potential multicollinearity. Bootstrapping with 5,000 resamples was employed to generate 95% bias-corrected confidence intervals (CI). An effect was considered statistically significant if the confidence interval did not include zero. All model parameters, including regression coefficients, standard errors, and t-values, were calculated using the default settings of the PROCESS macro. Statistical significance was set at a two-tailed *p*-value of <0.05.

## 3 Results

### 3.1 Sample characteristics

A total of 840 questionnaires were distributed. After excluding responses that did not meet the inclusion and exclusion criteria, 742 remained. A further five responses with a completion time of less than 3 min were removed, resulting in a final analytic sample of 737 participants (response rate: 87.7%). The sample comprised 87 physicians (11.8%), 196 midwives (26.6%), 332 nurses (45%), and 122 yuesao (16.6%) (Table 1). Mean age of participants was 37.3 ± 9.1 years, with an average of 10.9 ± 8.5 years of experience in their current positions. Most participants were married (74.4%) and had one or two children (69.2%). Regarding employment type, 33.9% held permanent positions, 27.1% were on short-term contracts, and 38.9% were on long-term contracts. Most were employed in public hospitals (82.8%), while 10.7% worked in private hospitals or home care organized through the hospital, and 6.5% in community hospitals.

**TABLE 1:** Participant characteristics.

Variables	M ± SD/*n* (%)	Presenteeism (M ± SD)	*r*/*t*/*F*	*P*-value
Age				
	37.3 ± 9.1	15.1 ± 4.2	−0.05	0.19
Gender				
Female	730 (99.1)	15.1 ± 4.2	1.25	0.21
Male	7 (0.9)	13.1 ± 3.4	–	–
Ethnicity				
Han	701 (95.1)	15.1 ± 4.2	−0.42	0.67
Minority	36 (4.9)	15.4 ± 4.4	–	–
Education experience				
≤12 years	124 (16.8)	14.6 ± 4.2	1.59	0.11
>12 years	613 (83.2)	15.2 ± 4.2	–	–
Marital status				
Married	548 (74.4)	15 ± 4.3	1.92	0.06*
Unmarried	189 (25.6)	15.6 ± 3.9	–	–
Children				
None	227 (30.8)	15.6 ± 3.9	4.07	0.017*
1	348 (47.2)	15.2 ± 4.3	–	–
2+	162 (22)	14.4 ± 4.3	–	–
Employment status				
Short-term contract	200 (27.1)	15.2 ± 3.8	3.09	0.05*
Long-term contract	287 (38.9)	14.7 ± 4.1	–	–
Permanent	250 (33.9)	15.6 ± 4.6	–	–
Monthly income				
≤6,000	116 (15.7)	15.5 ± 3.7	0.62	0.60
6,001–10,000	333 (45.2)	14.9 ± 4.1	–	–
10,001–20,000	256 (34.7)	15.3 ± 4.5	–	–
>20,000	32 (4.3)	14.8 ± 4.3	–	–
Institution type				
Private hospital or home	79 (10.7)	14.4 ± 4	8.27	0.0003*
Public hospital	610 (82.8)	15.1 ± 4.2	–	–
Community	48 (6.5)	17.4 ± 4	–	–
Night shifts (weekly)				
0	203 (27.5)	15.3 ± 4.3	0.47	0.62
1–2	290 (39.3)	14.9 ± 4.3	–	–
>2	244 (33.1)	15.2 ± 4	–	–
Position role				
Doctor	87 (11.8)	15.6 ± 4.6	1.90	0.13
Midwife	196 (26.6)	14.8 ± 4.1	–	–
Nurse	332 (45)	15.4 ± 4.2	–	–
Yuesao	122 (16.6)	14.6 ± 4	–	–
Work model				
Alone	275 (37.3)	14.9 ± 4.1	–1.06	0.29
Group	462 (62.7)	15.3 ± 4.3	–	–
Work hours (weekly)				
0–40	260 (35.3)	14.8 ± 4.1	1.50	0.22
41–48	308 (41.8)	15.4 ± 4.1	–	–
>48	169 (22.9)	15.1 ± 4.5	–	–
Years of experience	10.9 ± 8.4	15.1 ± 4.2	−0.01	0.70

*r*, Pearson; *t*, student *t*-test; F, ANOVA, *p < 0.1.

### 3.2 Pearson correction analysis

Workload was positively correlated with both presenteeism (*r* = 0.19, *p* < 0.01) and compassion fatigue (*r* = 0.35, *p* < 0.01) (as shown in Table 2). Compassion fatigue was also positively correlated with presenteeism (*r* = 0.49, *p* < 0.01). In contrast, self-compassion was negatively correlated with workload (*r* = −0.20, *p* < 0.01), compassion fatigue (*r* = −0.44, *p* < 0.01), and presenteeism (*r* = −0.47, *p* < 0.01).

**TABLE 2:** Descriptive statistics and Pearson correlation analysis of workload, compassion fatigue, self-compassion, and presenteeism.

Variables	*M*	SD	1	2	3	4	5	6
1. Workload	63.3	15.91	1	–	–	–	–	–
2. Compassion fatigue	39.22	25.1	0.35**	1	–	–	–	–
3. CFSS burnout	25.47	16.27	0.37**	0.98**	1	–	–	–
4. CFSS STS	13.75	9.8	0.30**	0.94**	0.85**	1	–	–
5. Self-compassion	39.84	6.2	−0.20**	−0.44**	−0.44**	−0.39**	1	–
6. Presenteeism	15.13	4.21	0.19**	0.49**	0.50**	0.42**	−0.47**	1

**p < 0.01.

### 3.3 Mediation analysis

The results of the mediation analysis are presented in Figure 1 and Table 3. After adjusting for marital status, number of children, employment status, and institution type, workload showed a significant positive direct effect on presenteeism (Path c, B = 0.05, 95% CI [0.03, 0.07]), supporting Hypothesis 1: When compassion fatigue was included as a mediator, significant associations were found for Path a (workload → compassion fatigue: B = 0.54, 95% CI [0.43, 0.64]) and Path b (compassion fatigue → presenteeism: B = 0.08, 95% CI [0.07, 0.09]). This indicates that higher workload was linked to increased compassion fatigue, which in turn predicted greater presenteeism. The significant indirect effect (B = 0.04, 95% CI [0.03, 0.06]) confirms compassion fatigue as a full mediator (Hypothesis 2).

**TABLE 3:** Mediation model.

Model summary	R^2^	*F*	B	SE	*t*	95% CI	*P*-value
Total effect							
Path c, workload → presenteeism	0.07	7.89	0.05	0.01	5.133	0.031, 0.069	0.001
Direct effect							
Path a, workload → compassion fatigue	0.15	18.6	0.535	0.056	9.576	0.425, 0.644	0.001
Path b, compassion fatigue → presenteeism	0.26	32.58	0.08	0.006	13.82	0.069, 0.091	0.001
Path c’, workload → presenteeism	–	–	0.008	0.009	0.812	−0.011, 0.026	0.417
Indirect effect							
Path a*path b	–	–	0.043	0.007	–	0.029, 0.057	–

Covariates: marital status, children, employment status, institution type.

### 3.4 Moderated mediation analysis

**FIGURE 2:**
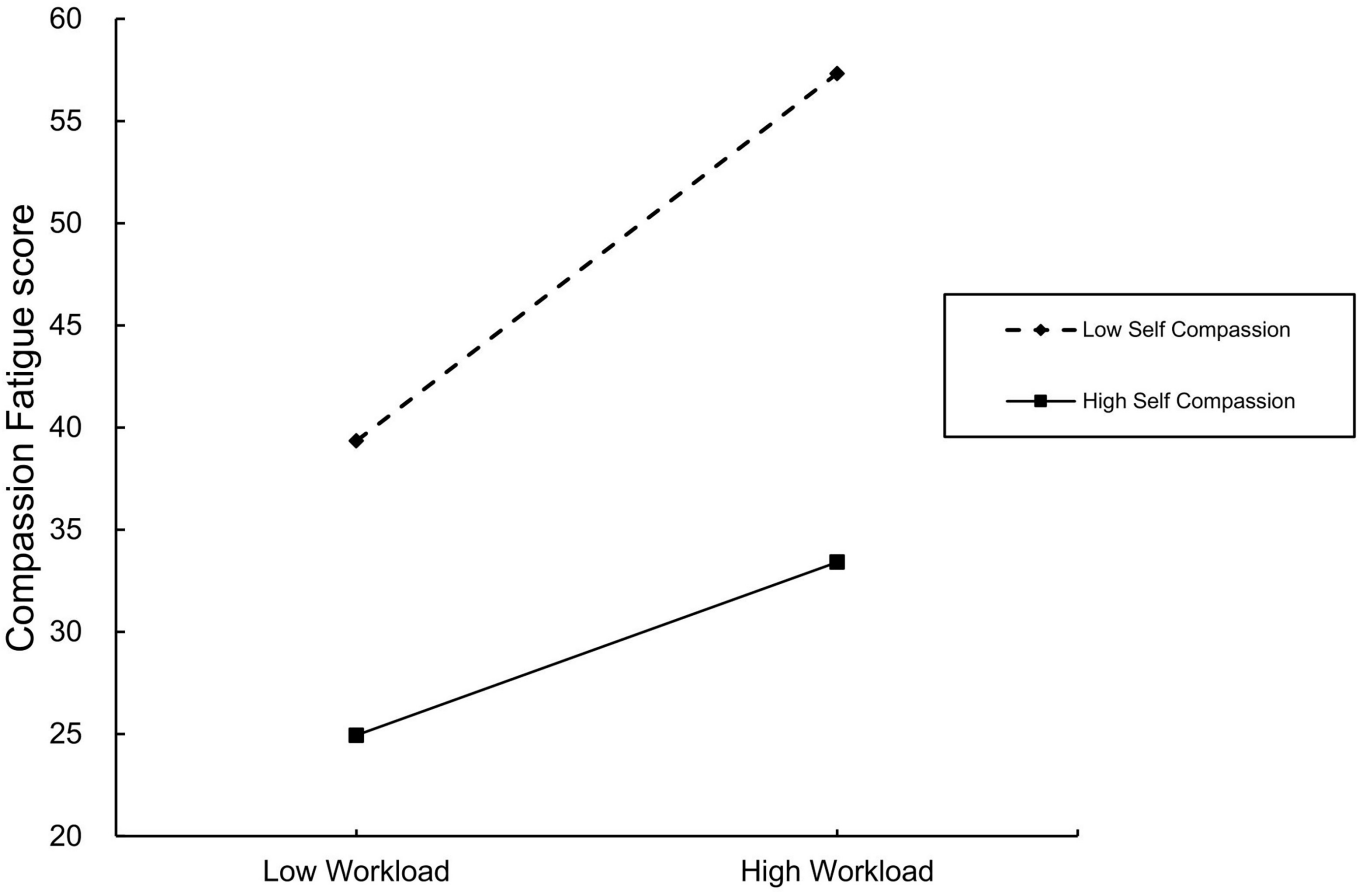
Simple slope test.

Results of the moderated mediation analysis are presented in Figure 2 and Table 4. The interaction between workload and self-compassion on compassion fatigue was significant (B = −0.02, 95% CI [−0.04, −0.01]), indicating that self-compassion moderated this relationship, which supports Hypothesis 3. Simple slope analyses (Supplementary Table 1) revealed that the positive association between workload and compassion fatigue was strongest at low levels of self-compassion (M−1SD: B simple = 0.60, 95% CI [0.43, 0.70]), moderate at the mean level (B = 0.42, 95% CI [0.31, 0.52]), and weakest at high levels of self-compassion (M+1SD: B = 0.27, 95% CI [0.13, 0.41]). Interpreted within scale ranges (self-compassion: 12–60; compassion fatigue: 13–130). This pattern confirms that higher self-compassion buffers the impact of workload on compassion fatigue.

**TABLE 4:** Moderated mediation model.

Model pathways	R^2^	*F*	B	SE	*t*	95% CI
Workload → compassion fatigue	0.29	33.02	0.416***	0.052	7.973	0.313, 0.518
Self-compassion → compassion fatigue	–	–	−1.546***	0.131	−11.797	−1.803, −1.289
Workload * self-compassion → compassion fatigue	–	–	−0.024**	0.008	−3.116	−0.039, −0.009
Workload → presenteeism	0.26	32.58	0.008	0.009	0.812	−0.011, 0.026
Compassion fatigue → presenteeism	–	–	0.080	0.006	13.82	0.069, 0.091

***P < 0.001, **P < 0.01.

Furthermore, bias-corrected percentile bootstrap analysis confirmed that the conditional indirect effects of workload on presenteeism through compassion fatigue were significant under different levels of self-compassion, and the index of the moderated mediation was significant (B = −0.002, 95% CI [−0.003, −0.001]), supporting Hypothesis 4 (Supplementary Table 2). This indicates that the strength of the indirect effect of workload on presenteeism (via compassion fatigue) varied meaningfully with self-compassion. Specifically, high self-compassion substantially attenuated—and in practical terms, nearly neutralized—this mediated pathway.

**FIGURE 3:**
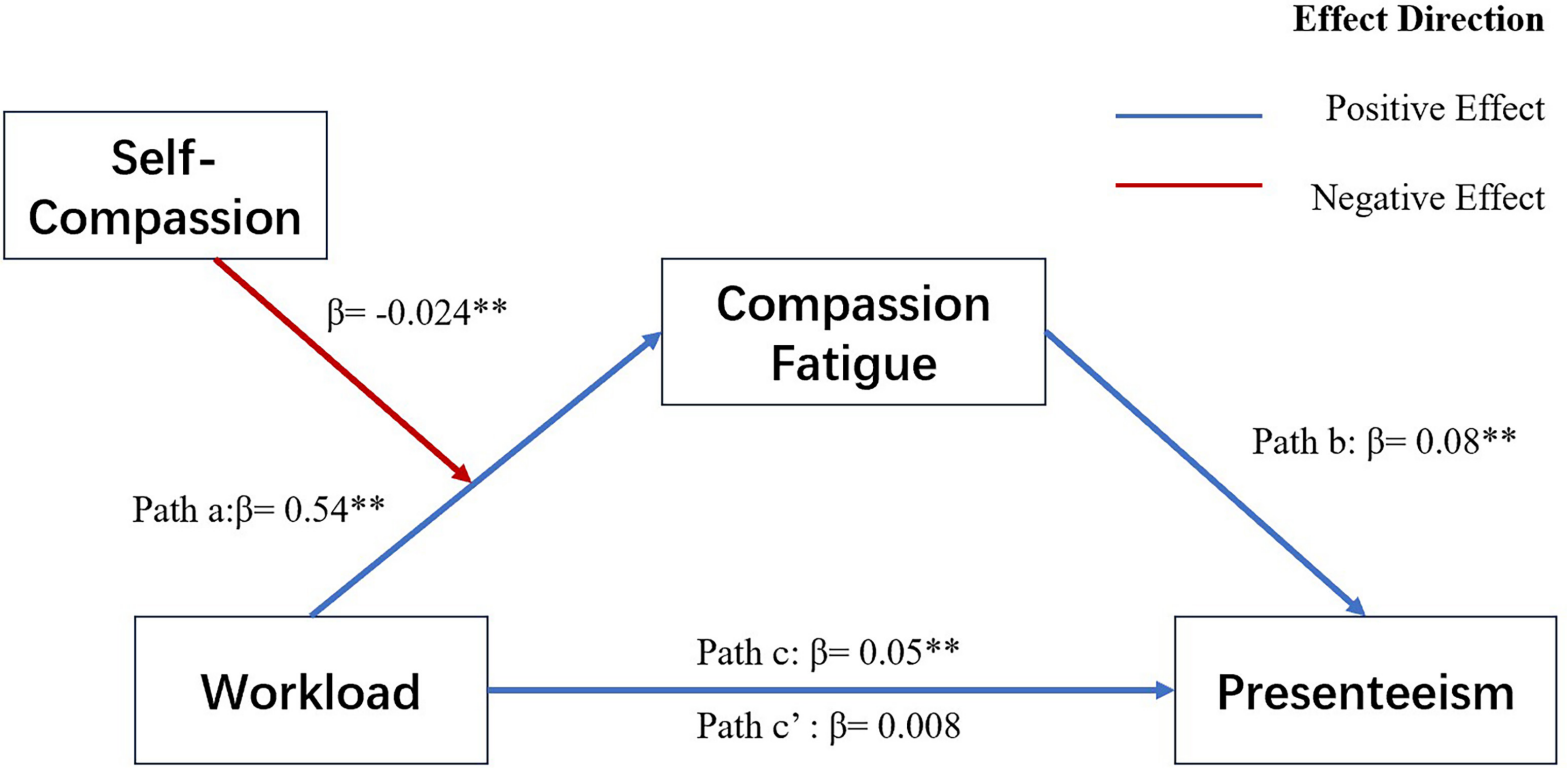
A moderated mediation model. Path coefficients are shown. Path a, work overload’s impact on compassion fatigue; Path b, compassion fatigue’s impact on presenteeism; Path c, total effect of work overload on presenteeism; Path c’, direct effect of work overload on presenteeism when compassion fatigue as a mediator; **Represents a significance level of *p* < 0.01.

Figure 3 illustrates the pathway of the moderated mediation model. The path coefficients showed that all relationships in the model were significant, except for the direct effect. After including compassion fatigue as a mediator and self-compassion as a moderator, the direct effect of workload on presenteeism was no longer significant. Therefore, the relationship between workload and presenteeism was fully mediated by compassion fatigue, and the association between workload and compassion fatigue was moderated by self-compassion.

## 4 Discussion

To the best of our knowledge, this is the first study to examine the roles of compassion fatigue and self-compassion in the relationship between workload and presenteeism among MNH professionals. Our findings revealed that workload was significantly associated with presenteeism, that compassion fatigue mediated this relationship, and that self-compassion moderated the pathway between workload and compassion fatigue.

### 4.1 Relationship between workload and presenteeism

In our sample, MNH professionals reported moderately high levels of workload (63.30 ± 15.91) and presenteeism (15.13 ± 4.21). These levels are higher than those found in studies of nurses and physicians working in non-maternal and newborn units (Kong et al., 2025), highlighting the particular severity of presenteeism in this workforce and underscoring the need for targeted attention.

Consistent with Hypothesis 1, we found a significant positive relationship between workload and presenteeism, indicating that MNH professionals with heavier workloads are more likely to attend work despite physical or psychological strain, a finding aligned with broader literature on presenteeism antecedents (Jiang et al., 2023). This association can be understood within the specific context of MNH care. Professionals in this field often contend with persistent staffing shortages, especially in high-volume settings serving older or high-risk pregnant women (Nove et al., 2021). These demands amplify both the psychological and physical workload of MNH professionals, thereby contributing to presenteeism (Qu et al., 2022; Tan et al., 2025). Given its potential to compromise provider wellbeing, organizational efficiency, and patient safety, targeted interventions are warranted (Matthews et al., 2024).

### 4.2 The mediating role of compassion fatigue

Our study confirms Hypothesis 2, demonstrating that compassion fatigue mediates the relationship between workload and presenteeism. Specifically, heavier workloads predicted greater compassion fatigue, which in turn predicted a higher likelihood of presenteeism This mediation pathway is well-supported by Conservation of Resources (COR) theory. Sustained high workload acts as a chronic demand that depletes essential psychological resources, leading to compassion fatigue, characterized by psychological exhaustion and reduced capacity to care (Bakker and Demerouti, 2024). This finding aligns with evidence that the intense psychological burden in healthcare renders professionals vulnerable to such fatigue, diminishing their coping abilities and work performance (Erbe, 2022).

Consequently, interventions aimed at mitigating presenteeism must proactively address compassion fatigue. To counteract the resource depletion process, hospital administrators should implement participatory and flexible scheduling to ensure adequate rest, alongside clarifying clinical roles and fostering collaboration to reduce burdens from unclear boundaries (Epstein et al., 2023). Furthermore, establishing on-call or casual staffing arrangements to accommodate fluctuations and unpredictability in workload demands can help alleviate the burden on core clinical staff (Olmstead et al., 2014). In addition, systematic fatigue screening, such as daily self-reports or wearable-based monitoring of sleep, activity and stress, can help early identification of maternal and child health professionals at high risk of working while ill, allowing timely implementation of targeted recovery strategies, including structured short breaks during shifts and regular rotations away from high-intensity clinical duties (Ito-Masui et al., 2025; Kang and Hur, 2026; Xiang et al., 2025). In parallel, structured support programs such as mindfulness meditation, interactive group seminars, and resilience training, may reduce compassion fatigue and indirectly lower presenteeism (Pollock et al., 2020).

### 4.3 The moderating role of self-compassion

This study revealed that self-compassion moderated both direct and indirect effects between workload and presenteeism. Specifically, higher levels of self-compassion attenuated the positive association between workload and presenteeism, both directly and indirectly by buffering its effect on compassion fatigue. From a theoretical perspective, this buffering effect can be understood within the JD-R framework, where self-compassion functions as a personal resource (Erbe, 2022). Higher self-compassion was associated with a weaker relationship between workload and compassion fatigue, indicating a stable buffering effect. This aligns with evidence that self-compassion is linked to lower burnout and better stress regulation (Willems et al., 2021). By fostering psychological stability and reducing maladaptive self-criticism, self-compassion helps professionals preserve their capacity for empathy and engagement, mitigating the exhaustion that leads to presenteeism and supporting compassionate care (Neff, 2023).

Furthermore, the moderated mediation analysis showed that the indirect association between workload and presenteeism through compassion fatigue was weaker at higher levels of self-compassion. In practical terms, high self-compassion did not just reduce but nearly neutralized this detrimental chain of effects. Importantly, a qualitative study highlighted that professionals’ willingness to engage in self-compassion often requires “permission” both from themselves and from others in their broader environment, including leadership, organizational culture, and even patients (Andrews et al., 2020).

Therefore, efforts must be multi-level. At the individual and educational level, self-compassion should be positioned as a core professional competency and integrated into both pre-licensure and continuing education for MNH professionals (Musseri Navon et al., 2025). This involves challenging the traditional, self-sacrificing “patient-first” ideal by affirming that effective caregiving necessitates self-care. Evidence from a study of Australian community nurses suggests that a positive working environment is associated with reduced emotional labor and lower levels of stress-related presenteeism and job neglect (Karimi et al., 2017). At the organizational and managerial level, leaders must actively create psychologically safe and supportive work environments that legitimize and foster self-compassion (Pérez-Castejón et al., 2025). This includes providing access to psychological support, demonstrating humanistic leadership, and implementing structured programs such as mindfulness-based training, group yoga, or cognitive-behavioral therapy (Lamothe et al., 2018; Ofei-Dodoo et al., 2020), which have proven effective in enhancing self-compassion

## 5 Limitations

This study has several limitations. First, its cross-sectional design precludes causal inferences regarding relationships among workload, compassion fatigue, self-compassion, and presenteeism. Future longitudinal or mixed-methods research is needed to verify these temporal dynamics and gain deeper insight into the lived experiences of MNH professionals. Second, all data were collected via self-report measures, which may be subject to social desirability and recall biases, potentially influencing the accuracy of findings. Third, despite recruiting participants from 13 hospitals of varying levels (including general and specialized maternal-child health facilities) to enhance diversity, the sample was drawn solely from a single metropolitan region in China. This limits the generalizability of results to rural areas or settings with more constrained healthcare resources. Additionally, the pilot test included only six maternal and child healthcare professionals, although their feedback was sufficient to assess the clarity, readability, and comprehensibility of the questionnaire, the small sample size remains a limitation. Future studies could expand the pilot sample to further optimize the measurement tool.

## 6 Conclusion

This study identified a high level of presenteeism among maternal and newborn health professionals in China and elucidated key psychological processes underlying this phenomenon. Workload was associated with presenteeism both directly and, more significantly, indirectly through compassion fatigue, highlighting the role of psychological and emotional strain in translating high job demands into impaired work functioning. Crucially, self-compassion moderated this process, weakening the indirect association between workload and presenteeism via compassion fatigue.

These results provide a clear, psychologically informed and multi-faceted intervention blueprint. Healthcare administrators can mitigate presenteeism not only by managing excessive workload and addressing compassion fatigue but also, and perhaps most proactively, by strategically fostering self-compassion as a potent, modifiable personal resource. Specific actionable strategies include: (1) integrating brief self-compassion training into routine staff development programs; (2) establishing structured peer support groups to reduce compassion fatigue; and (3) implementing workload monitoring systems with adjustable staffing ratios during high-acuity shifts. Implementing such integrated strategies can enhance psychological wellbeing, improve job performance, and ultimately support the delivery of higher-quality maternal-newborn healthcare.

## Data Availability

The raw data supporting the conclusions of this article will be made available by the authors, without undue reservation.

## Footnotes

1
https://www.wjx.cn
