# Supplementary material for: Association between workload, compassion fatigue and presenteeism among maternal and newborn health professionals: the moderated role of self-compassion
Source: Front Psychol. 2026 Jun 15;17:1793586. doi: 10.3389/fpsyg.2026.1793586 (PMC13310782; doi:10.3389/fpsyg.2026.1793586)
Supplement: Supplementary file 1 [file Supplementary_file_1.docx]

**Table S1. The impact of workload on compassion fatigue at different of self-compassion**

|  | B | SE | t | 95%CI | p |
| --- | --- | --- | --- | --- | --- |
| Self-compassion |  |  |  |  |  |
| M-1SD | 0.565 | 0.07 | 8.066 | 0.427, 0.703 | 0.001 |
| M | 0.416 | 0.052 | 7.973 | 0.313, 0.518 | 0.001 |
| M+1SD | 0.266 | 0.072 | 3.717 | 0.126, 0.407 | 0.001 |

Note: 95%CI: LLCI, ULCI.

**Table S2. The conditional indirect effects of Workload->Compassion Fatigue->Presenteeism at different of self-compassion**

|  | Indirect effect | BootSE | Boot LL 95% CI | Boot UL 95% CI |
| --- | --- | --- | --- | --- |
| Self-compassion |  |  |  |  |
| M-1SD | 0.045 | 0.010 | 0.026 | 0.064 |
| M | 0.033 | 0.007 | 0.020 | 0.046 |
| M+1SD | 0.021 | 0.005 | 0.012 | 0.031 |
| the index of mediated moderation | -0.002 | 0.001 | -0.003 | -0.001 |
